# Optimization of deep eutectic solvent extraction of *Piper nigrum* L. and evaluation of hepatoprotective effects against acute ethanol-induced liver injury in mice

**DOI:** 10.3389/fphar.2026.1933093

**Published:** 2026-09-10

**Authors:** Jia-Lu Wang, Xing-Xing Liu, Xin-Yu Li, Chuan-Gui Wang, Hong-Yu Wang, Jing-Han Jia, Yu-Yue Luo, Xin-Hua Song, Chao Wang, Wen-Long Sun

**Affiliations:** 1 School of Life Sciences and Medicine, Shandong University of Technology, Zibo, China; 2 Shandong Engineering Research Center of Precision Nutrition and Healthy Aging, Qilu Medical University, Zibo, China; 3 Research Institute of Natural Products and Health Industry Innovation, Qilu Medical University, Zibo, China

**Keywords:** alcohol-associated liver disease, deep eutectic solvents, gut microbiota, hepatoprotective effect, *Piper nigrum* L., piperine

## Abstract

**Introduction:**

Alcohol-associated liver disease (ALD) represents a growing global health burden, for which safe and accessible therapeutic options remain limited. As a widely used medicinal and food plant, Piper nigrum L. contains piperine as its core hepatoprotective alkaloid. However, conventional organic solvent extraction poses environmental risks, leaves solvent residues, and causes loss of synergistic endogenous bioactives. As sustainable green extraction media, deep eutectic solvents (DESs) show great potential for phytochemical enrichment, yet their impacts on the hepatoprotective potency and gut microbiota-regulatory activity of black pepper extracts remain unclear.

**Methods:**

A choline chloride-1,4-butanediol-based ultrasonic-assisted DES extraction system was optimized via single-factor experiments and Box-Behnken design. We systematically compared the phytochemical profiles, in vitro antioxidant activities, and in vivo hepatoprotective efficacies of DES extracts versus conventional 60% ethanol extracts using a mouse model of acute ethanol-induced liver injury, and employed 16S rRNA gene sequencing to analyze gut microbiota composition.

**Results:**

Under the optimized conditions (extraction time of 60 min, water content of 30%, solid-to-liquid ratio of 1:28 g/mL, molar ratio of choline chloride to 1,4-butanediol of 1:3, ultrasonic power of 400 W, and temperature of 40 °C), the DES process achieved a piperine yield of 44.40 ± 0.84 mg/g. In the DPPH radical scavenging assay, DES extract exhibited better in vitro antioxidant activity than the ethanol extract. In mice with acute ethanol-induced liver injury, DES extract reduced hepatic lipid accumulation, oxidative stress, and hepatocellular injury. 16S rRNA sequencing revealed that DES extract treatment was correlated with altered gut microbial composition under ethanol stimulation, including decreased relative abundance of Muribaculaceae and Clostridia UCG-014, and increased relative abundance of Lactobacillus murinus.

**Discussion:**

Collectively, these findings indicate that DES-derived black pepper extract is associated with favorable hepatoprotective phenotypes in the acute ethanol-induced liver injury model, alongside distinct correlational changes in gut microbiota composition. The study provides technical support for green high-value processing of plant-based spice resources, and scientific insights for developing microbiota-targeted plant-derived functional foods.

## Graphical Abstract

**Figure:**
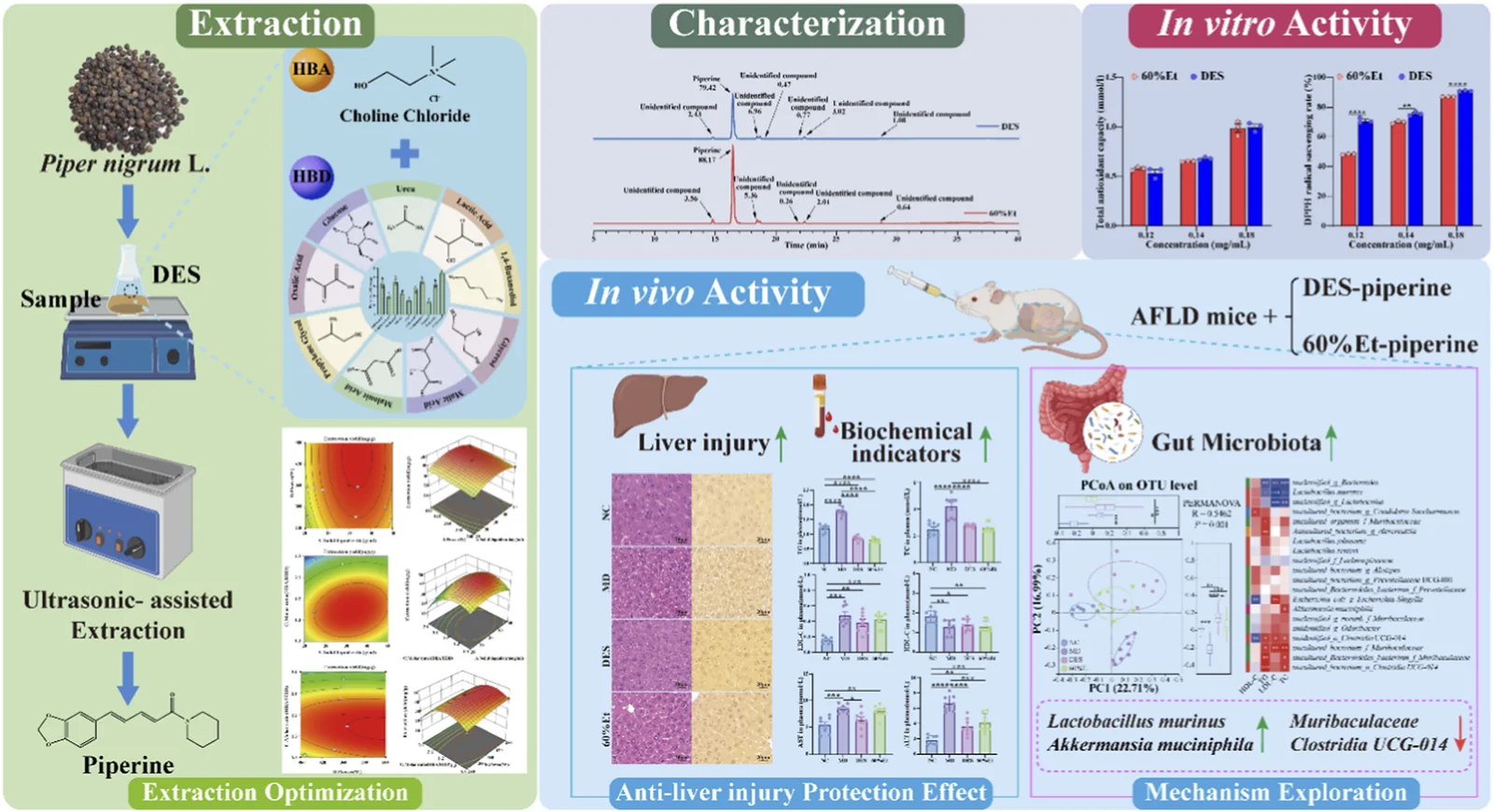

## 1 Introduction

Chronic excessive alcohol intake is the primary cause of alcohol-associated liver disease (ALD), a widespread metabolic liver disease that has emerged as a major global public health issue. The pathogenesis of ALD is closely associated with excessive hepatic lipid accumulation, disrupted oxidative stress homeostasis and impaired gut-liver axis balance (Tan et al., 2025). Without effective intervention, ALD can further progress into liver fibrosis, cirrhosis, and even hepatocellular carcinoma, posing a serious threat to human health (Rehm and Shield, 2019). Currently approved pharmacological interventions for ALD remain limited by suboptimal efficacy, adverse effects, and high costs, highlighting an urgent need for safe, accessible, and effective preventive and therapeutic strategies.

In this context, bioactive metabolites derived from medicinal and food plants have emerged as promising candidates for liver disease management owing to their multi-targeted actions, favorable safety profiles, and long history of dietary use. Dietary intervention with plant-derived bioactive represents a promising strategy for ALD prevention (Sharma et al., 2022). As one of the most widely consumed spices worldwide, *Piper nigrum* L. and its major alkaloid piperine have been documented to possess antioxidant, anti-inflammatory, and gut microbiota-regulating activities, and show great potential for functional food development (Shityakov et al., 2019; Quijia and Chorilli, 2020; Cao et al., 2024). Piperine has been reported to regulate lipid metabolism and exert antioxidant effects (Du et al., 2020; Raghunath et al., 2025). However, traditional organic solvent-based extraction techniques for natural products suffer from inherent drawbacks, including toxic and volatile solvents usage, severe safety hazards, undesirable solvent residues, low extraction efficiency and excessive energy consumption. Most conventional extraction solvents are non-degradable, which fails to comply with green chemistry principles and sustainable industrial development requirements (Chemat et al., 2012).

As a new generation of eco-friendly and sustainable solvents, deep eutectic solvents (DESs) are formed via self-assembly of hydrogen bond donors and acceptors. These systems exhibit excellent solubilization capacity for phytochemicals, good biocompatibility, and low environmental impact (Choi and Verpoorte, 2019; Ferreira and Sarraguça, 2024). Our research group previously established a choline chloride-malic acid DES extraction system for flavonoids from *Lophatherum gracile* Brongn. and verified its ALD activity via gut microbiota regulation (Luo et al., 2026). Nevertheless, the extraction behavior and bioactive characteristics of DESs vary drastically among plant matrices and target compounds. While DESs have shown outstanding advantages in plant alkaloid extraction (Ivanović et al., 2020; Dheyab et al., 2021), systematic optimization of DES-based piperine extraction processes, and their effects on the gut microbiota-modulating activity and *in vivo* bioactivity of pepper extracts, remain largely unexplored. Accumulating evidence confirms that gut microbiota dysbiosis drives the onset and progression of ALD: ethanol exposure disrupts intestinal barrier integrity, induces overgrowth of pathobionts, and promotes translocation of bacterial endotoxins into the liver via the portal vein, further aggravating hepatic inflammation and injury (Gao, 2024; Shen et al., 2025). Therefore, targeting gut microbiota regulating represents a promising mechanism for evaluating the hepatoprotective effects of plant-derived bioactives.

Accordingly, this study aimed to optimize a choline chloride-1,4-butanediol-based ultrasonic-assisted DES extraction process for piperine, and to compare the phytochemical characteristics and bioactivities of DES extracts with conventional 60% ethanol extracts. We further evaluated their hepatoprotective effects in an acute ethanol-induced liver injury mouse model and explored potential underlying mechanism from the perspective of gut microbiota regulating. This work provides technical support for green high-value processing of plant-based spices, and scientific insights for developing microbiota-targeted plant-derived functional foods.

## 2 Materials and methods

### 2.1 Plant material

Dried ripe fruits of *P*. *nigrum* L. were purchased from Beijing Tongrentang Co., Ltd. The botanical identity was authenticated by a pharmacognosy expert, and a voucher specimen was deposited at the School of Life Sciences and Medicine, Shandong University of Technology. The raw materials were oven-dried at 50 °C until a constant weight was achieved, then ground and passed through a 50-mesh screen. The resulting powder was kept in a sealed container under cool, dry conditions for later extraction procedures.

### 2.2 Chemicals and reagents

Choline chloride (RG), malonic acid (AR), propylene glycol (AR), oxalic acid (AR), glucose (AR), urea (AR), lactic acid (AR), 1,4-butanediol (AR), glycerol (AR), and malic acid (AR) were obtained from Shanghai Macklin Biochemical Co., Ltd. Piperine reference standard (purity ≥98%) was purchased from Aladdin Co., Ltd. Commercial assay kits for DPPH free radical scavenging capacity (DPPH), 2,2′-Azinobis-(3-ethylbenzothiazoline-6-sulfonate (ABTS), Alanine aminotransferase (ALT), Aspartate aminotransferase (AST), Total cholesterol (TC), Triglycerides (TG), High-density lipoprotein cholesterol (HDL-C), Low-density lipoprotein cholesterol (LDL-C), Malondialdehyde (MDA), Superoxide dismutase (SOD) and Glutathione peroxidase (GSH-Px) were sourced by Nanjing Jiancheng Bioengineering Institute. A BCA protein assay kit was supplied by Beijing ComWin Biotech Co., Ltd.

### 2.3 Preparation of DES

Prior to use, choline chloride and all hydrogen bond donors were thoroughly dried to prevent moisture uptake. DESs were formulated by combining HBAs and HBDs at a molar ratio of 1:1, followed by continuous magnetic stirring at 80 °C until a clear, transparent, and viscous liquid formed. Deionized water was then added to obtain a water content of 30% (w/w) to modulate the water content of the DES systems, and the resulting solutions were homogenized by stirring for later use (Smith et al., 2014).

### 2.4 Conventional ethanol-based ultrasound-assisted extraction of piperine

First, 0.2 g of *P*. *nigrum* L. powder was weighed into a 10 mL centrifuge tube, and 60% ethanol (ethanol: water = 6:4) was added (Martinez-Sena et al., 2017). The mixture was then subjected to ultrasonic treatment at 40 °C and 100 W for 30 min. Subsequently, the mixture was centrifuged at 10,000 rpm/min for 10 min, and the supernatant was collected as the black pepper ethanol extract.

### 2.5 DES-based ultrasound-assisted extraction of piperine

Briefly, 0.2 g of black pepper powder was accurately weighed and combined with 6.0 mL of DES solution. The mixture was vortexed for 1 min to achieve uniform dispersion, followed by ultrasonic extraction at 100 W and 40 °C for 30 min. The mixture was transferred to a 50 mL centrifuge tube and centrifuged at 4 °C at 10,000 rpm for 10 min to collect the supernatant. The obtained supernatant was further centrifuged at 13,000 rpm for 10 min. Subsequently, 1 mL supernatant was transferred into a 10 mL centrifuge tube and diluted 10-fold with 60% ethanol. The dilution not only reduced the high viscosity of DES but also adjusted the analyte concentration to fit the linear range of the standard curve. Finally, the absorbance was measured at 343 nm with reference to the established standard curve (Cvjetko Bubalo et al., 2016). The extraction parameters described in this section were only applied for preliminary screening of optimal DES solvent formulations.

### 2.6 Post-extraction processing

After optimized ultrasonic DES extraction, the supernatant was loaded onto an AB-8 macroporous adsorption resin column. The column was first rinsed with ultrapure water to fully elute residual choline chloride and 1,4-butanediol, followed by gradient ethanol-water elution to collect piperine-rich fractions. Combined eluates were concentrated under reduced pressure at 45 °C via rotary evaporation to remove ethanol. The concentrated solution was transferred into a MD44–100 dialysis bag (MWCO 100 Da) and dialyzed against ultrapure water at 4 °C for 24 h, with ultrapure water refreshed 2–3 times daily to further remove residual DES components (Dai et al., 2025). After dialysis, the aqueous retentate retained inside the dialysis bag was collected.

The supernatant obtained from 60% ethanol ultrasonic extraction was directly concentrated to complete dryness via rotary evaporation at 45 °C without resin purification and dialysis steps. The dried solid residue was reconstituted with ultrapure water to form a homogeneous aqueous suspension for unified lyophilization treatment.

The aqueous retentate from the DES group and the reconstituted aqueous suspension of ethanol extract residue were separately pre-frozen at −20 °C overnight to avoid splashing during lyophilization. Both samples were vacuum-lyophilized at −60 °C under high vacuum for 12 h to obtain lyophilized crude extracts. A single independent batch of powder was prepared for each extraction group. All subsequent phytochemical quantification, *in vitro* antioxidant assays and *in vivo* animal intervention trials were performed exclusively using these two batches of lyophilized extracts.

### 2.7 Standard curve establishment and yield calculation

Accurately weighed piperine reference standard was fully dissolved in 60% ethanol and transferred into a 100 mL volumetric flask, followed by volume adjustment with 60% ethanol to prepare the stock solution. The stock solution was diluted gradually to obtain standard working solutions with concentrations of 0.5, 1, 3, 5, and 7 μg/mL. The absorbance of each standard solution was determined at 343 nm. Linear regression was performed with piperine concentration as the abscissa and absorbance as the ordinate (Dai et al., 2013). The calibration curve equation was thus obtained as $y=0.1544x-0.0291,R^{2}=0.9988$ .

The extraction yield of piperine was computed using the formula below:

$$\text{Extraction yield of piperine }\left(mg/g\right)=\frac{C\times V\times N}{M}$$

In this formula, C represents the piperine concentration in the sample solution, V is the final constant volume of the extract, N corresponds to the dilution multiple, and M is the mass of the black pepper powder used.

### 2.8 Single-factor experimental optimization

Single-factor experiments were carried out to investigate the effects of six key parameters on piperine extraction yield, including the water content of the DES (10%–50%), solid-to-liquid ratio (1:10-1:50 g/mL), ultrasonic power (120–600 W), extraction time (15–75 min), HBA-HBD molar ratio (1:1-3:1), and extraction temperature (40 °C–80 °C) on the extraction yield were investigated (Cvjetko Bubalo et al., 2016). The single-variable control method was adopted in each group, and other parameters were kept fixed when a single factor was investigated.

### 2.9 Response surface methodology optimization

Based on single-factor results, the extraction temperature and DES moisture content were maintained at 40 °C and 30%, respectively, throughout the RSM experiments, solid-to-liquid ratio, ultrasonic power, and HBA-HBD molar ratio was selected as the three dominant influencing factors. A three-factor, three-level Box-Behnken design (BBD) was adopted, using piperine extraction yield as the response value. Seventeen experimental trials were arranged to evaluate the interaction effects of the main factors and identify the optimal extraction conditions (Bezerra et al., 2008). The quadratic polynomial regression model was established as follows:

$$Y=\beta_{0}+\sum_{i=1}^{\;3}\beta_{i}X_{i}+\sum_{i=1}^{\;3}\beta_{ii}X_{i}^{2}+\sum_{i=1}^{\;2}\sum_{j=i+1}^{\;3}\beta_{ij}X_{i}X_{j}$$

where Y denotes the response yield, β_0_ is the intercept term, βᵢ, βᵢᵢ, and β_*ij*_ are the regression coefficients for the linear, quadratic, and interactive effects, respectively, and Xᵢ and X_j_ signify the independent variables.

### 2.10 HPLC qualitative analysis

Qualitative and quantitative analysis of piperine was carried out on a Shimadzu LC-20AT HPLC instrument fitted with a UV detector. A Morchem Caprisil C18-B column (250 mm × 4.6 mm, 5 μm) was employed for chromatographic separation. The mobile phase comprised water (A) and methanol (B) delivered at 1.0 mL/min, with the gradient program set as: 50% B at 0–5.01 min; 50%–55% B at 5.01–10.00 min; 55%–58% B at 10.00–12.00 min; 58%–60% B at 12.00–14.00 min; 60%–63% B at 14.00–16.00 min; 63%–65% B at 16.00–18.00 min; 65%–100% B at 18.00–35.00 min; 100%–50% B at 35.00–35.01 min; and held at 50% B from 35.01 to 45.01 min. The UV detection wavelength was set at 352 nm, with an injection volume of 20 μL. Prior to injection, all sample solutions were filtered through a 0.45 μm organic membrane. HPLC was used only for qualitative chromatographic comparison of extract compositions, and was not applied for piperine quantitation in this study.

### 2.11 MS qualitative analysis

MS analysis was performed for compound identification. The chromatographic separation was achieved on an Atlantis dC18 column (150 × 4.6 mm, 5 μm; Waters, USA). The mobile phase consisted of (A) 0.1% formic acid in water and (B) 0.1% formic acid in acetonitrile, with isocratic elution using 65% B for 0–5 min. The flow rate was 0.75 mL/min, the injection volume was 10 μL, and the UV detection wavelength was set at 352 nm. For mass spectrometric detection, electrospray ionization (ESI) in positive ion mode was employed, and data acquisition was performed in full-scan mode over the mass range of *m/z* 90-900. The ion source temperature was maintained at 300 °C, with a nebulizing gas flow of 1.5 L/min and a drying gas flow of 15 L/min. Piperine was identified by comparison with an authentic standard. The raw data were processed using Thermo Scientific Compound Discoverer 3.0 software for compound identification and peak annotation.

### 2.12 *In vitro* antioxidant activity evaluation

Free radical scavenging capacities of DES-extract and conventional ethanol-extract were evaluated via DPPH and ABTS assays according to the standard kit instructions.

### 2.13 Animal experiment design

Six-week-old male Kunming mice were obtained from Shandong Pengyue Laboratory Animal Co., Ltd. All procedures involving animals were reviewed and approved by the Animal Experiment Ethics Committee of Shandong University of Technology (Approval No. YLX202509021). Mice were raised under standard conditions (20 °C–24 °C, 40%–70% humidity, 12 h light/dark cycle) with free access to food and water. Animals were randomly assigned to four groups (n = 8): normal control (NC) group, model (MD) group, black pepper DES extract (DES) group, and black pepper ethanol extract (60%Et) group. Mice in the MD, DES, and 60%Et groups were given 52% ethanol (0.01 mL/g) by gavage daily to induce acute ethanol-induced liver injury. The DES and 60%Et groups were simultaneously treated with corresponding crude black pepper extracts at 50 mg/kg (based on the weight of crude extract) for 9 days, while the NC and MD groups were given an equal volume of distilled water (Xie et al., 2025).

At the end of intervention, all mice were fasted for 8 h. After anesthesia with isoflurane, mice were euthanized by cervical dislocation. Blood samples were collected for plasma biochemical detection. Liver tissues were dissected, partially fixed with universal tissue fixative for histological observation, and the remaining tissues were frozen rapidly and stored at −80 °C. Colonic tissues were fixed with Carnoy’s fixative for subsequent analysis. Fecal samples were collected and frozen in liquid nitrogen for 16S rRNA sequencing.

### 2.14 Biochemical assays

Plasma concentrations of ALT, AST, TC, TG, HDL-C and LDL-C, along with SOD, MDA and GSH-Px levels in both plasma and liver tissues, were strictly determined following the operating instructions of corresponding commercial kits.

### 2.15 Liver histopathological examination

Fixed liver samples in advance were embedded in paraffin wax and sectioned continuously at a thickness of 3 μm. Hematoxylin-eosin (H&E) staining and modified Sirius Red staining were performed to observe hepatic morphological changes and lipid deposition under an optical microscope.

### 2.16 16S rRNA gene sequencing and gut microbiome analysis

Microbial genomic DNA was isolated from fecal samples, and the 338F/806R primer set was employed to amplify the target sequences. High-throughput sequencing was performed on the Pacbio Sequel IIe platform (Shanghai Majorbio Bio-Pharm Technology Co., Ltd.). Subsequent bioinformatics processing was performed via the Majorbio Cloud platform (https://cloud.majorbio.com). Alpha diversity metrics, including Chao and Ace indices, were computed using Mothur software (v1.30.2), with inter-group comparisons evaluated by the Kruskal–Wallis H test. Beta diversity was assessed using Bray-Curtis distance. LEfSe analysis was applied to identify differentially abundant microbial taxa, and Spearman’s rank correlation was employed to examine associations between key gut bacterial populations and hepatic biochemical parameters.

### 2.17 Statistical analysis

All data are presented as mean ± standard error of the mean (SEM). GraphPad Prism 10.1.2 was employed for statistical evaluations. Inter-group comparisons were conducted using one-way ANOVA and two-way ANOVA coupled with Tukey’s multiple comparison *post hoc* test, with statistical significance set at *p < 0.05, **p < 0.01, ***p < 0.001, ****p < 0.0001. For correlation analysis, Spearman’s rank correlation coefficients were calculated between microbial taxa and clinical parameters. The Benjamini–Hochberg false discovery rate method was applied to correct for multiple testing, and adjusted *q-values were used to determine statistical significance (*q < 0.05, **q < 0.01, ***q < 0.001, ****q < 0.0001).

## 3 Results

### 3.1 Screening of optimal DES systems for piperine extraction

**FIGURE 1:**
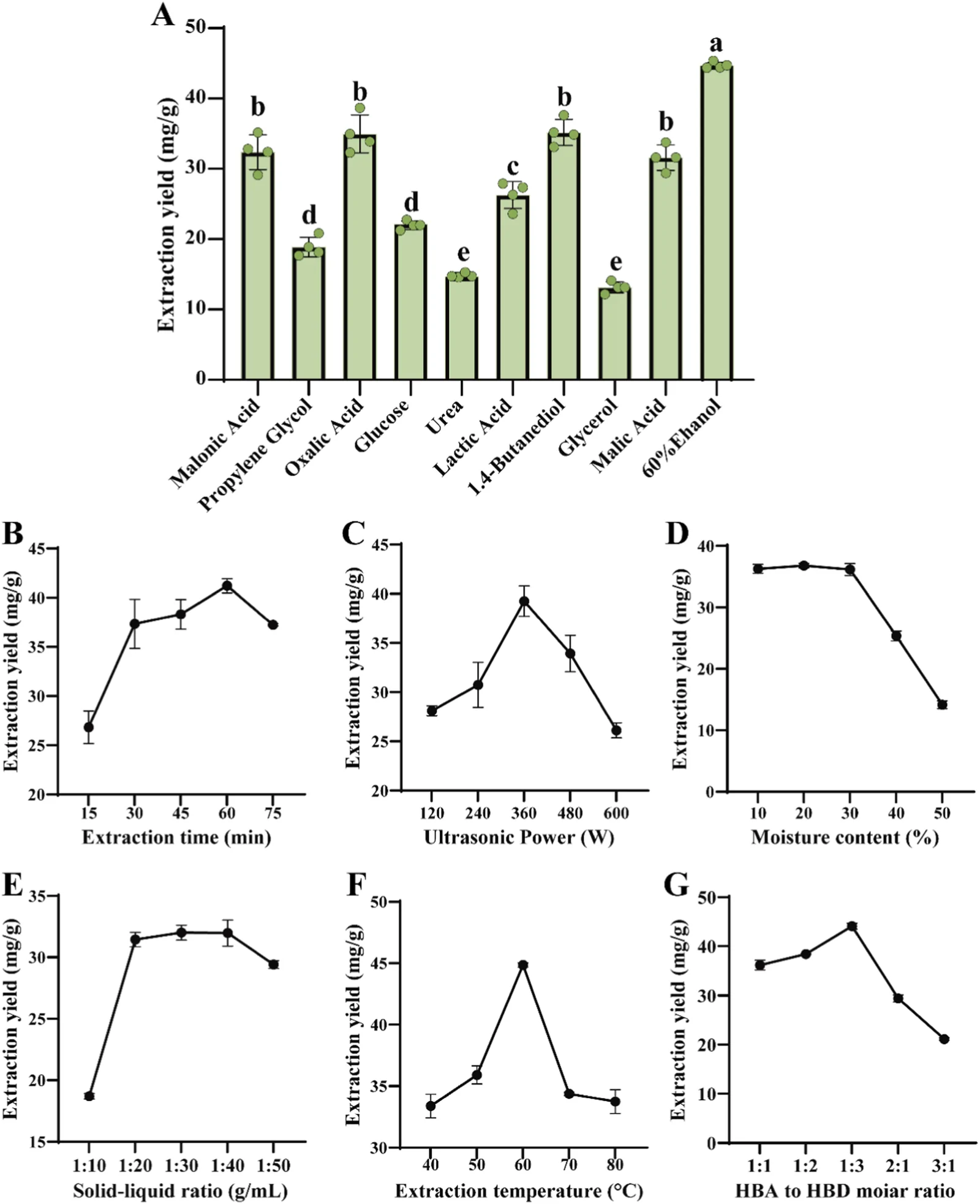
Effects of different extraction parameters on piperine extraction yield. **(A)** Screening of optimal DES formulations; **(B)** Extraction time; **(C)** Ultrasonic power; **(D)** Moisture content; **(E)** Solid-to-liquid ratio; **(F)** Extraction temperature; **(G)** HBA to HBD molar ratio. Data are expressed as mean ± SEM from three independent experiments. Different lowercase letters indicate significant differences among groups (*p* < 0.05) by one-way ANOVA followed by Tukey’s test.

Nine choline chloride-based DES formulations were prepared and tested for their effectiveness in ultrasound-assisted piperine extraction from black pepper, with 60% ethanol as the traditional reference solvent. Extraction efficiency depended strongly on the DES type. Carboxylic acid-based DESs generally outperformed their nitrogen-containing and sugar-based counterparts in terms of piperine yield. Among all candidates, the choline chloride-1,4-butanediol system yielded the numerically highest piperine content (Figure 1A). Although this system and several carboxylic acid-based DESs, including the oxalic acid group, showed no statistically significant difference in extraction yield, the final selection was not based on extraction efficiency alone. A systematic evaluation of viscosity, stability, safety, and cost was therefore conducted. The carboxylic acid-based DESs presented high viscosity, poor homogeneous stability, and concerns regarding corrosiveness and irritation due to their acidity. In contrast, the choline chloride-1,4-butanediol DES offered moderate viscosity, stable single-phase behavior, low toxicity, weak corrosiveness, and low-cost raw materials. Taking these factors together, the choline chloride-1,4-butanediol DES was selected as the extraction medium for subsequent process optimization.

### 3.2 Single-factor analysis of extraction parameters

To identify the appropriate ranges of key operational variables for piperine extraction using the selected DES, six critical parameters, including extraction time, ultrasonic power, DES moisture content, solid-to-liquid ratio, extraction temperature, and HBA-HBD molar ratio, were investigated to clarify their individual effects on piperine extraction yield.

Extraction time exhibited a positive effect on piperine yield within 15–60 min, and the maximum yield was obtained at 60 min. Excessively prolonged extraction (75 min) caused a slight decline in piperine content, which might be attributed to the oxidative degradation of active ingredients under long-term ultrasonic treatment (Figure 1B). Ultrasonic power significantly affected extraction efficiency: the yield increased gradually from 120 W to 360 W and reached the peak at 360 W, whereas further power elevation (480–600 W) markedly reduced the yield, possibly because of structural degradation of piperine induced by excessive ultrasonic energy (Figure 1C). DES moisture content showed a distinct inhibitory effect on extraction efficiency. A relatively high and stable yield was maintained at low moisture levels (0%–20%), while moisture content exceeding 30% sharply decreased the piperine yield, which dropped below 10 mg/g at a moisture content of 50% (Figure 1D). For the solid-to-liquid ratio, the extraction yield increased rapidly in the range of 1:10-1:20 g/mL and remained stable at a high level with further solvent addition (1:20-1:50 g/mL), indicating that sufficient solvent dissolution was achieved at a moderate solid-to-liquid ratio (Figure 1E). Extraction temperature positively promoted piperine extraction within 40 °C–60 °C, with the yield rising from approximately 32 mg/g to over 43 mg/g at 60 °C. Continuous temperature elevation (70 °C–80 °C) significantly reduced the yield, which was probably caused by the thermal decomposition of piperine (Figure 1F). The HBA-HBD molar ratio also exerted a pronounced effect: the optimal ratio of choline chloride to 1,4-butanediol was 1:3, and any deviation from this ratio led to an obvious reduction in extraction yield (Figure 1G). Based on the single-factor results, solid-liquid ratio, ultrasonic power, and HBA-to-HBD molar ratio were selected as independent variables for RSM optimization. These three factors showed the strongest and most distinct effects on extraction yield, each presenting a clear optimal point that warranted further investigation. In contrast, moisture content exerted negligible influence within the tested range and was therefore fixed at an appropriate level. Extraction temperature was also excluded, as its effect was relatively moderate compared to the selected factors; fixing this condition helped avoid potential thermal degradation and simplified the experimental design.

### 3.3 Process optimization via Box-Behnken response surface methodology

According to the single-factor experimental trends, three key influential parameters, namely, solid-to-liquid ratio (A), ultrasonic power (B), molar ratio (C) were further optimized via RSM. The experimental design and corresponding piperine yields are listed in Table 1. Significant differences were observed among different experimental combinations, and several groups exhibited higher yields than single-factor experiments, suggesting notable interactive effects among variables and the necessity of multi-factor optimization.

**TABLE 1:** Box-Behnken experimental design and piperine extraction yields.

Run	A (solid-to-liquid ratio)	B (ultrasonic power)	C (molar ratio)	Extraction yield (mg/g)
	g/mL	W	HBA/HBD	
1	20	360	2:1	12.6036
2	30	240	2:1	22.7137
3	30	360	1:3	43.1153
4	40	240	1:3	37.4870
5	30	240	1:2	32.8562
6	30	360	1:3	43.3873
7	30	360	1:3	44.3976
8	30	360	1:3	44.2422
9	30	480	1:2	40.7448
10	30	480	2:1	24.5401
11	20	480	1:3	35.2979
12	20	240	1:3	32.8108
13	40	360	1:2	31.6839
14	40	360	2:1	23.8601
15	20	360	1:2	33.0440
16	40	480	1:3	39.7668
17	30	360	1:3	44.3199

The quadratic polynomial regression equation fitted by Design Expert 13 software was as follows:

$$\begin{array}{cc} \text{Extraction yield }\left(\text{mg}/g\right)= & 43.89+2.34A\\ & +1.81B-6.79C-0.0518AB\\ & +3.23AC-1.52BC-6.27A^{2}-1.28B^{2}-12.40C^{2} \end{array}$$

ANOVA demonstrated that the regression model was highly significant (*p* < 0.0001), whereas the lack of fit was not significant (*p* = 0.1454), indicating accurate model fitting without systematic error (Table 2). The high coefficient of determination (R^2^ = 0.9965) and adjusted R^2^ (0.9919), coupled with a low coefficient of variation (2.45%) verified the excellent accuracy, stability, and predictive ability of the regression model. According to the ANOVA results, the AC interaction (solid-liquid ratio × HBA/HBD molar ratio) was the strongest (F = 60.67, *p* < 0.0001), followed by BC (power × molar ratio) (F = 13.33, *p* = 0.0082), whereas the AB interaction (solid-liquid ratio × power) was not significant (F = 0.0156, *p* = 0.9041). Therefore, the interaction strength is ranked as follows: AC > BC > AB.

**TABLE 2:** ANOVA results for the fitted regression model.

Source	Sum of squares	Degree of freedom	Mean square	*F*-value	*P*-value (Prob > F)
Model	1,362.24	9	151.36	219.63	<0.0001
A	43.83	1	43.83	63.60	<0.0001
B	26.22	1	26.22	36.63	0.0005
C	368.51	1	368.51	534.72	<0.0001
AB	0.0107	1	0.0107	0.0156	0.9041
AC	41.81	1	41.81	60.67	0.0001
BC	9.19	1	9.19	13.33	0.0082
A^2^	165.70	1	165.70	240.44	<0.0001
B^2^	6.88	1	6.88	9.99	0.0159
C^2^	647.43	1	647.43	939.44	<0.0001
Residual	4.82	7	0.6892	​	​
Lack of fit	3.40	3	1.13	3.20	0.1454
Pure error	1.42	4	0.3548	​	​
Cor total	1,367.06	16	​	​	​

**FIGURE 2:**
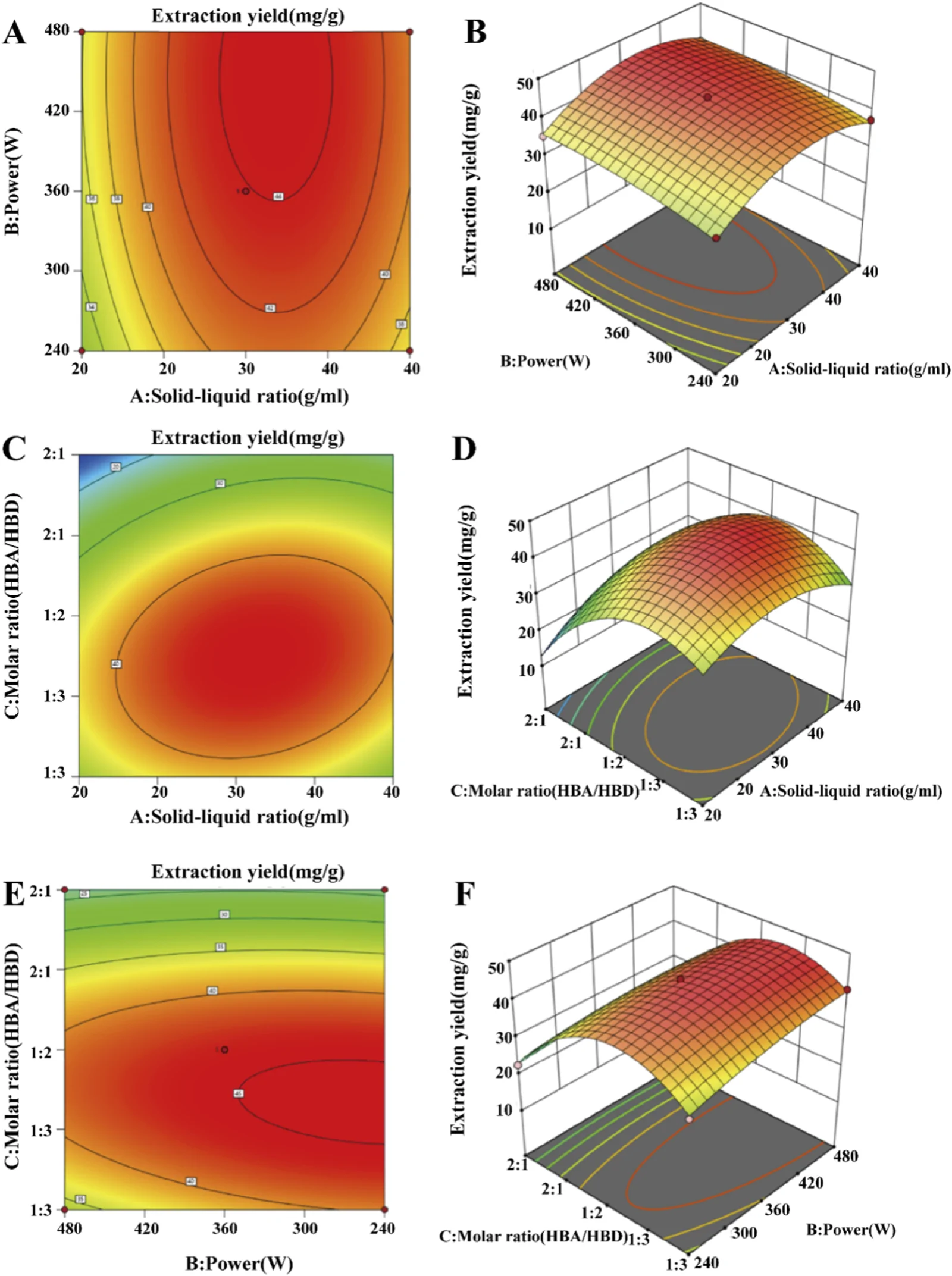
Contour and 3D response surface plots of factor interactions affecting piperine extraction yield. **(A,B)** Interaction between solid-to-liquid ratio and ultrasonic power; **(C,D)** Interaction between solid-to-liquid ratio and molar ratio; **(E,F)** Interaction between ultrasonic power and molar ratio.

According to the quadratic model, the optimal extraction conditions were a solid-to-liquid ratio of 1:28.4235 g/mL, a molar ratio (HBA/HBD) of 1:3, and a power of 399.252 W. Under these conditions, the predicted maximum extraction yield was 44.9628 mg/g. However, considering the operational precision of laboratory equipment, these parameters were adjusted to practically feasible values: a solid-to-liquid ratio of 1:28 g/mL and a power of 400 W (the molar ratio of 1:3 was retained as an integer). As shown in Figure 2A, the contour lines near the optimum (power 360–420 W, solid-liquid ratio 25–35 g/mL) are relatively sparse and the color gradient is uniform, suggesting that the response surface is flat in this region. This confirms that the extraction process is robust to small parameter fluctuations. To evaluate the reproducibility of the optimized extraction process, six independent verification experiments were conducted under the optimal conditions. The obtained piperine yield was 44.40 ± 0.84 mg/g (n = 6), with a relative standard deviation (RSD) of 1.89%. The relative deviation between the experimental mean value and the model-predicted value was 1.25%. These six independent runs covered the full workflow of sample weighing, DES solvent preparation, ultrasonic extraction, sample pretreatment and UV-Vis measurement. The low RSD demonstrates good consistency between experimental and predicted results, as well as the excellent reproducibility and reliability of the established DES extraction process.

### 3.4 Compositional differences and *in vitro* antioxidant activity

**FIGURE 3:**
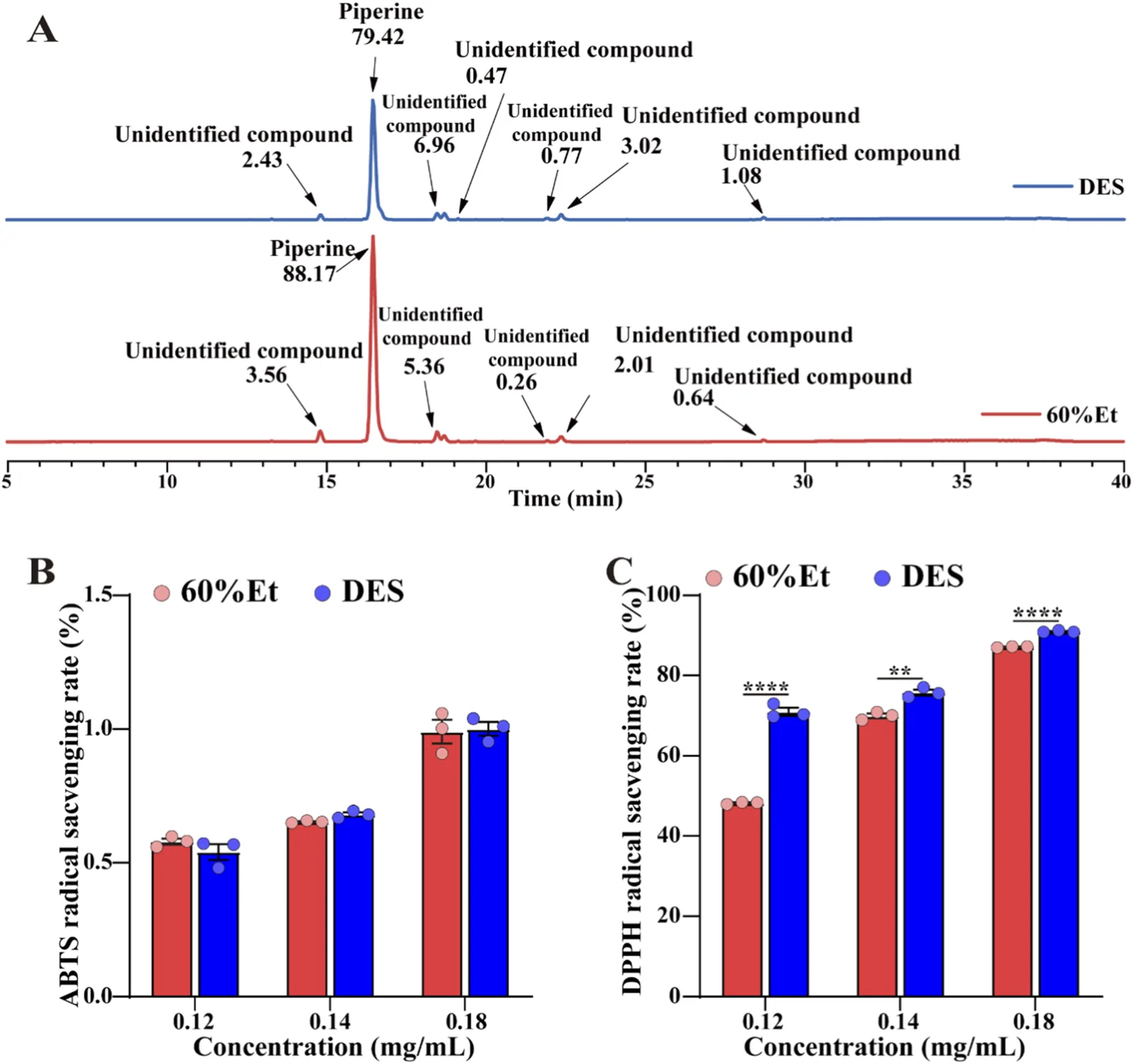
Comparison of compositional profiles and antioxidant activities between DES and 60% ethanol extracts. **(A)** HPLC-UV chromatograms (Shimadzu LC-20AT, C18-B column, 352 nm) of DES and 60% ethanol crude extracts; **(B)** ABTS radical scavenging activity; **(C)** DPPH radical scavenging activity. Data are presented as mean ± SEM from three independent replicates. Data were analyzed by two-way ANOVA with Tukey’s *post hoc* test. *p < 0.05, **p < 0.01, ***p < 0.001, ****p < 0.0001.

HPLC analysis was performed to compare the component differences between DES-extracted and conventional 60% ethanol-extracted black pepper products. The piperine peak area accounted for 79.42% of the total chromatographic area in the DES extract. For the 60% ethanol extract, the relative proportion of piperine increased to 88.17%, accompanied by a reduced abundance of miscellaneous components, indicating higher piperine relative content in ethanol extracts (Figure 3A). It should be noted that these area percentage values are normalized from LC-MS total ion chromatograms and are intended for qualitative comparison of peak profiles only; they do not represent absolute purity or content, and cannot be used for quantitative comparison between samples.

*In vitro* DPPH and ABTS radical scavenging assays were further conducted to evaluate the antioxidant capacity of the two extracts. Both extracts exhibited concentration-dependent antioxidant activity, achieving the highest scavenging activity at 0.18 mg/mL. Two-way ANOVA showed that for the DPPH assay, DES extract exhibited significantly higher scavenging activity than 60% ethanol extract, with significant main effects of method and concentration and a significant interaction between them (*P* < 0.0001 for all). For the ABTS assay, no significant difference was observed between the two extracts (Figures 3B,C). These results revealed that DES extract presented distinct *in vitro* DPPH radical scavenging performance compared with the ethanol extract under the tested conditions.

### 3.5 DES extract ameliorates plasma lipid disorders and liver injury *in vivo*

**FIGURE 4:**
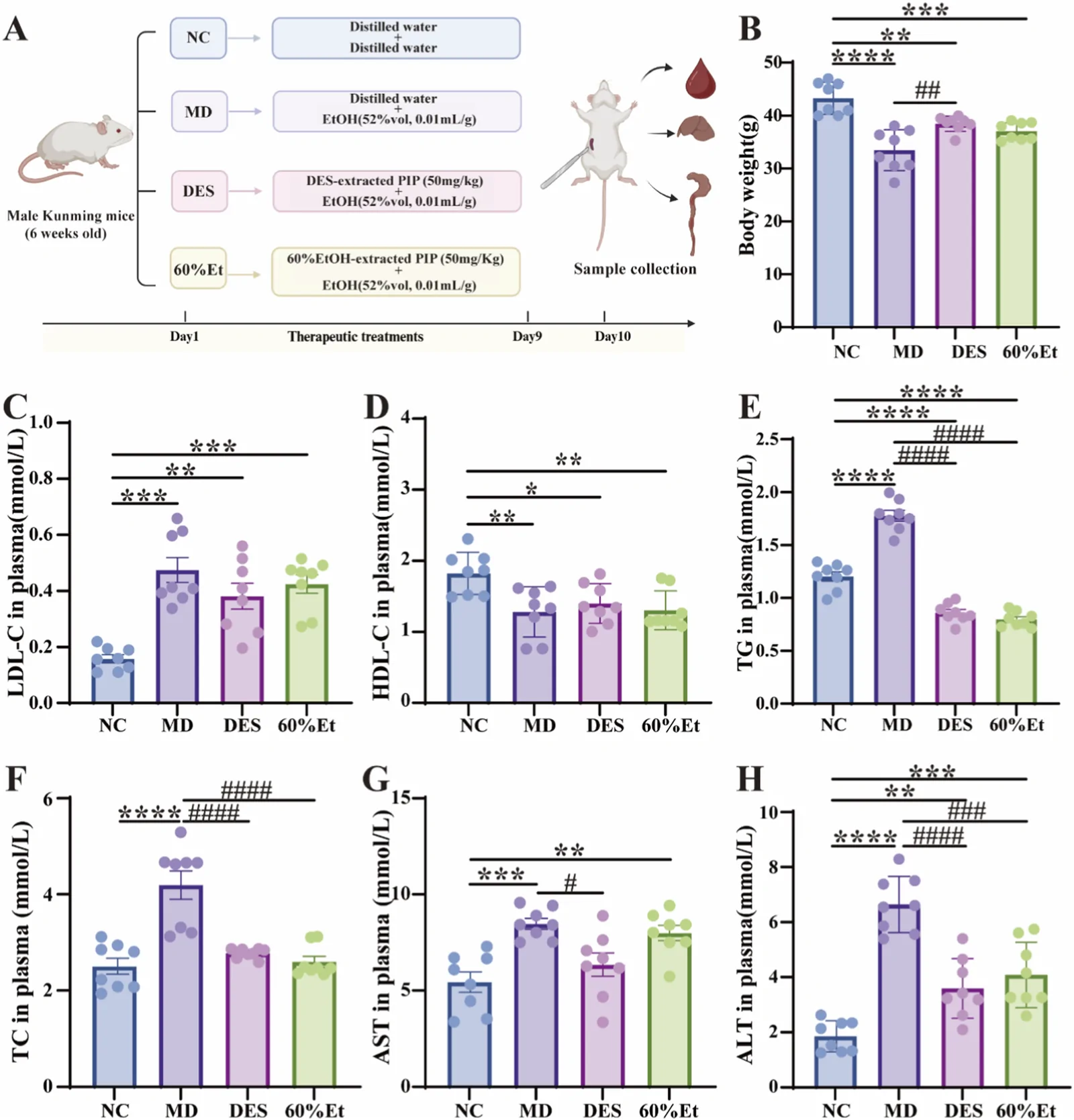
Effects of DES and 60% ethanol extracts on plasma lipid profiles and liver function in acute ethanol-induced liver injury mice. **(A)** Schematic diagram of the animal experimental design; **(B)** Mouse body weight; **(C)** Plasma LDL-C level; **(D)** Plasma HDL-C level; **(E)** Plasma TG level; **(F)** Plasma TC level; **(G)** Plasma AST level; **(H)** Plasma ALT level. Data are shown as mean ± SEM (n = 8). *p < 0.05, **p < 0.01, ***p < 0.001, ****p < 0.0001 vs. NC group; ^#^ *p* < 0.05, ^##^ *p* < 0.01, ^###^ *p* < 0.001, ^####^ *p* < 0.0001 vs. MD group.

The regulatory actions of black pepper DES extract and black pepper ethanol extract on plasma lipid parameters, hepatic fat accumulation, and oxidative stress indices were subsequently assessed in acute ethanol-induced liver injury mice. A schematic representation of the animal study is provided in Figure 4A. Following ethanol administration, the MD group showed a marked decrease in final body weight when compared with the NC group. The DES extract significantly alleviated ethanol-induced body weight loss (*p <* 0.01), while the ethanol extract showed no significant effect no restoring body weight (Figure 4B). Plasma lipid profiles and liver function biomarkers were further detected to validate the successful establishment of the acute ethanol-induced liver injury model and comprehensively compare the hepatoprotective effects of the two black pepper extracts. Relative to the healthy NC group, ethanol-treated mice developed typical acute ethanol-induced liver injury model, presenting obvious plasma metabolic disturbance and severe hepatocellular damage. Biochemical analysis confirmed that the MD group exhibited significantly elevated plasma LDL-C (*p* < 0.001), TG (*p* < 0.0001), and TC (*p* < 0.0001) levels, a marked reduction in plasma HDL-C (*p* < 0.01) level, and prominent upregulation of plasma AST (*p* < 0.001) and ALT (*p* < 0.0001) activities (Figures 4C–H). These consistent and significant biochemical abnormalities fully verified the successful induction of acute ethanol-induced liver injury in mice.

Black pepper extracted by two different extraction methods exhibited notable protective effects against ethanol-induced metabolic disorders and hepatic injury. Both extracts significantly reduced plasma TC (*p* < 0.0001) and TG (*p* < 0.0001), also attenuated the elevation of plasma ALT with distinct statistical significance for the two extraction approaches (*p <* 0.0001 and *p <* 0.001, respectively). For plasma AST, only the DES extract produced a statistically significant reduction (*p <* 0.05), while the ethanol extract merely caused a mild decreasing trend without statistical significance. Although both extracts slightly decreased LDL-C and moderately elevated HDL-C, these alterations did not reach statistical significance in either group (Figures 4C–H), which contributed to the amelioration of abnormal lipid metabolism and alleviation of hepatocellular damage. Both DES and 60% ethanol extracts of black pepper alleviated ethanol-induced disruptions to plasma lipid profiles relative to the MD group, consistent with the improved body weight of model mice (Figure 4B), Taken together, both extracts could ameliorate ethanol-induced disorders in plasma lipid and transaminase parameters. Notably, the DES extract showed differential regulatory effects on plasma ALT and AST levels relative to the conventional ethanol extract under the tested experimental conditions.

### 3.6 DES extract attenuates hepatic lipid accumulation and oxidative stress damage

**FIGURE 5:**
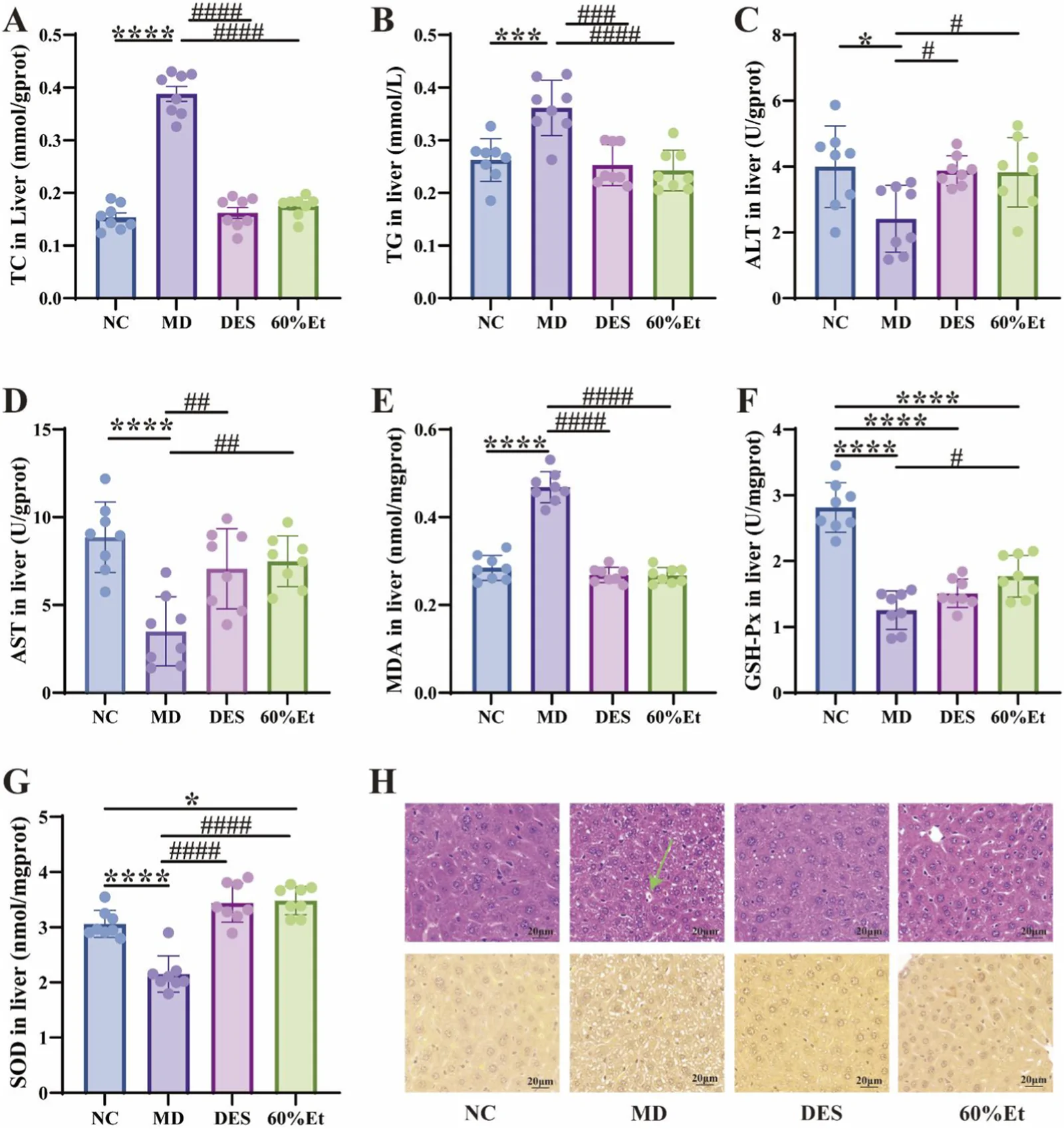
Effects of DES and 60% ethanol extracts on hepatic biochemical parameters and pathological changes in acute ethanol-induced liver injury mice. **(A)** Hepatic TC level; **(B)** Hepatic TG level; **(C)** Hepatic ALT level; **(D)** Hepatic AST level; **(E)** Hepatic MDA level; **(F)** Hepatic GSH-Px activity; **(G)** Hepatic SOD activity; **(H)** H&E and Sirius Red staining of liver tissues. Data are shown as mean ± SEM (n = 8). *p < 0.05, **p < 0.01, ***p < 0.001, ****p < 0.0001 vs. NC group; ^#^ *p* < 0.05, ^##^ *p* < 0.01, ^###^ *p* < 0.001, ^####^ *p* < 0.0001 vs. MD group.

Hepatic lipid accumulation and oxidative stress imbalance are core pathological features of acute ethanol-induced liver injury, which collectively exacerbate hepatic parenchymal injury. To further verify ethanol-induced hepatic damage and the protective effects of the two black pepper extracts, we systematically detected hepatic lipid metabolism, hepatocellular injury and oxidative stress levels. Compared with the NC group, acute ethanol-induced liver injury model mice showed severe hepatic metabolic dysfunction, characterized by markedly increased hepatic TC (*p* < 0.0001) and TG (*p* < 0.0001) contents (Figures 5A,B), as well as significantly downregulated hepatic ALT (*p* < 0.05) and AST (*p* < 0.0001) levels (Figures 5C,D), indicating obvious ethanol-triggered lipid deposition and hepatocellular damage. Notably, hepatic homogenate ALT and AST differ from leaked circulating plasma transaminases, as their activities are influenced by hepatocyte count, enzyme expression and metabolic conditions. We detected hepatic transaminases to reflect intrinsic hepatic metabolic status, providing supplementary information distinct from plasma enzyme indicators. Intervention with DES-extracted and 60% ethanol-extracted black pepper effectively alleviated abnormal hepatic lipid accumulation, reduced overexpressed hepatic transaminase levels, and ameliorated ethanol-induced hepatic metabolic disorder and hepatocellular injury in acute ethanol-induced liver injury mice.

Hepatic oxidative stress homeostasis was further evaluated by detecting oxidative and antioxidant biomarkers. The ethanol-treated MD group exhibited a significant increase in hepatic MDA content (*p* < 0.0001) (Figure 5E), a key lipid peroxidation indicator, accompanied by distinct reductions in the activities of the antioxidant enzymes SOD (*p* < 0.0001) and GSH-Px (*p* < 0.0001) (Figures 5F,G), confirming the induction of severe hepatic oxidative stress injury. Both black pepper extracts effectively reversed ethanol-induced oxidative damage by reducing excessive MDA accumulation (*p* < 0.0001) and restoring SOD activity (*p* < 0.0001), thereby stabilizing hepatic oxidative homeostasis and exerting favorable hepatoprotective effects in acute ethanol-induced liver injury mice.

Compared with the normal radiating hepatic plate architecture in the NC group, the MD group exhibited mild disorganization of hepatic plates and scattered widened interstitial gaps, indicating mild hydropic morphological damage induced by short-term acute ethanol administration. A representative disordered region is marked with an arrow in the MD H&E image. Both DES and 60% ethanol extract partially restored the regular hepatic cord structure (Figure 5H). Sirius Red staining showed no obvious collagen deposition across all groups, consistent with the short-term acute injury model, so lipid accumulation was interpreted mainly via hepatic TG and TC biochemical data instead of histological images.

### 3.7 DES extract regulates gut microbiota dysbiosis in acute ethanol-induced liver injury mice

**FIGURE 6:**
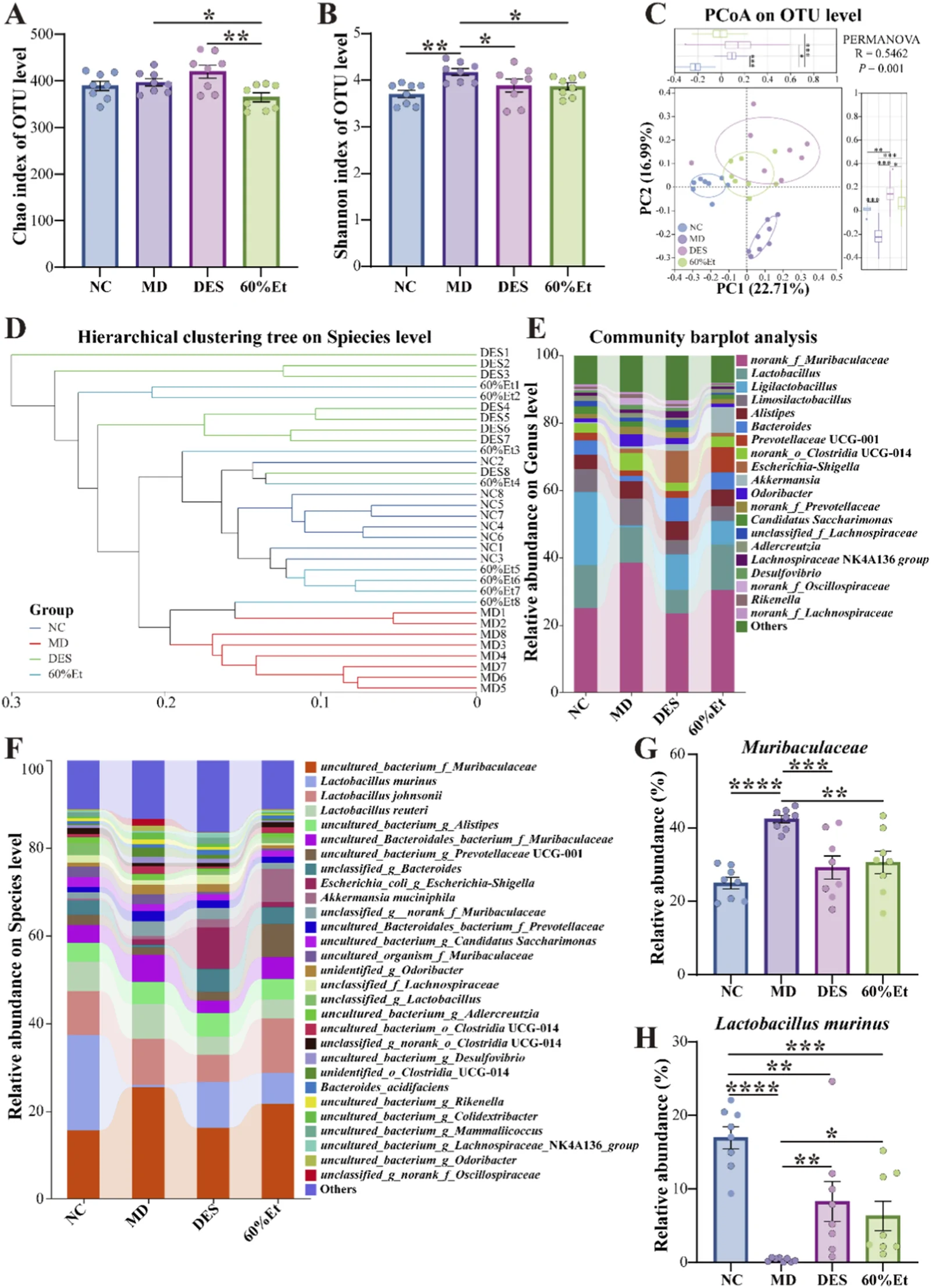
Effects of the two extracts on gut microbiota diversity and community structure in acute ethanol-induced liver injury mice. **(A)** Chao index based on Kruskal–Wallis H test; **(B)** Shannon index based on Kruskal–Wallis H test; **(C)** PCoA analysis at the OTU level; **(D)** Hierarchical clustering tree of gut microbiota at the species level; **(E)** Community bar plot of gut microbiota at the genus level; **(F)** Community heatmap of gut microbiota at the species level; **(G)** Relative abundance of *Muribaculaceae*; **(H)** Relative abundance of *Lactobacillus murinus*. Data are shown as mean ± SEM (n = 8). *p < 0.05, **p < 0.01, ***p < 0.001, ****p < 0.0001.

16S rRNA high-throughput sequencing was performed to evaluate the modulatory effects of the two extracts on gut microbiota composition in acute ethanol-induced liver injury mice. Alpha diversity analysis revealed that acute ethanol exposure disrupted gut microbial homeostasis in mice, as indicated by a significantly elevated Shannon index and a modest increase in the Chao index in the model group. Both black pepper extracts effectively attenuated the ethanol-induced abnormal rise in Shannon diversity; notably, DES extract significantly enhanced microbial richness, whereas the 60% ethanol extract reduced the Chao index to below normal levels (Figures 6A,B). PCoA based on Bray-Curtis distance revealed significant differences in gut microbiota community structure across groups (PERMANOVA, R = 0.5462, *p* = 0.001). The two treatment groups partially shifted away from the MD cluster; however, they did not fully converge with the NC region, indicating that neither extract completely restored the overall microbial community structure (Figure 6C). Community heatmap and hierarchical clustering analyses further showed that the DES group exhibited a clustering pattern relatively closer to the NC phenotype compared to the 60%Et group, suggesting a comparatively stronger modulatory potential (Figures 6D–F). At the taxonomic level, differential microbial taxa were analyzed across groups. The relative abundance of *Muribaculaceae* (family level)—which was markedly elevated in the model group—was suppressed by both extract treatments, with a more pronounced reduction observed in the DES group. Conversely, the abundance of *Lactobacillus murinus* (species level) was substantially depleted by ethanol exposure; both treatments partially restored its abundance, although no statistically significant difference was detected between the DES and 60%Et groups, and neither treatment returned its level to that of the NC group (Figures 6G,H).

**FIGURE 7:**
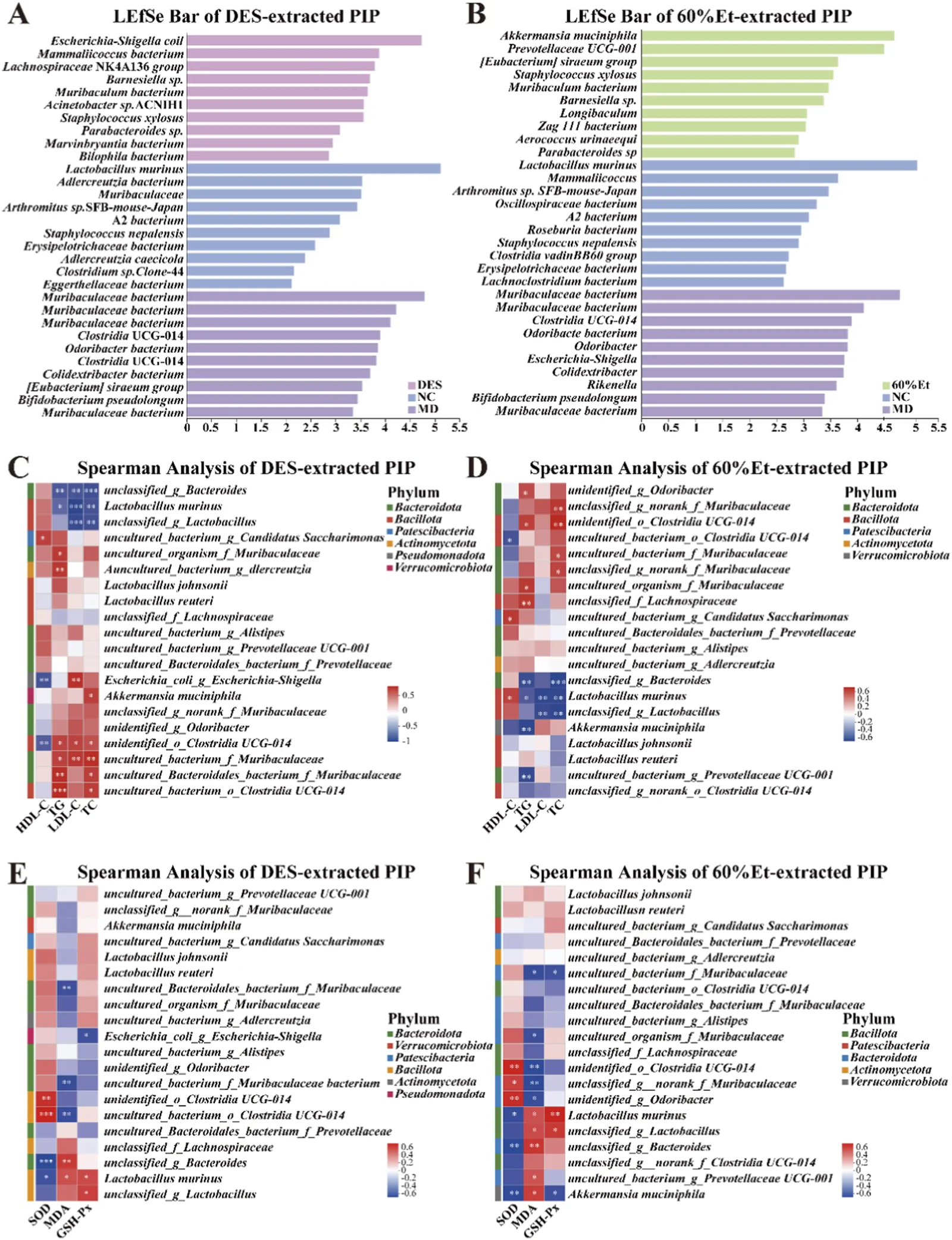
Effects of the two extracts on differential gut microbiota taxa and microbiota-biochemistry parameter correlation in acute ethanol-induced liver injury mice. **(A)** LEfSe bar plot showing differentially abundant bacterial taxa among the NC, MD and DES groups; **(B)** LEfSe bar plot showing differentially abundant bacterial taxa among the NC, MD, and 60% Et groups; **(C)** Spearman correlation between differential bacterial species and plasma lipid indices (HDL-C, TG, LDL-C, TC) in the DES group; **(D)** Spearman correlation between differential bacterial species and plasma lipid indices in the 60% Et group; **(E)** Spearman correlation between differential bacterial species and hepatic oxidative stress markers (SOD, MDA, GSH-Px) in the DES group; **(F)** Spearman correlation between differential bacterial species and hepatic oxidative stress markers in the 60% Et group. Data are presented as means ± SEM (n = 8) Correlation coefficients are represented by color intensity (red: positive; blue: negative). Significance was assessed using Spearman’s rank correlation test, with *P*-values adjusted for multiple comparisons via the Benjamini–Hochberg false discovery rate method. *q < 0.05, **q < 0.01, ***q < 0.001. Data are presented as means ± SEM (n = 8 per group).

LEfSe analysis (LDA threshold >2.0) was applied to identify key differential bacterial species driving gut microbiota dysbiosis in ethanol-induced ALD mice (Figures 7A,B). The results revealed obvious group-specific variations in microbial biomarker abundance. Specifically, the ethanol-exposed MD group showed a marked enrichment of *Muribaculaceae* and *Clostridia UCG-014* relative to the NC group, which were identified as dominant differential taxa closely associated with ethanol-induced gut microecological disturbance. Notably, the regulatory effects of the two extracts on the gut microbiota exhibited distinct characteristics. The DES extract intervention significantly reduced the ethanol-induced enrichment of *Muribaculaceae* and *Clostridia UCG-014*. In contrast, the 60% ethanol extract intervention specifically enriched *Akkermansia muciniphila*, but showed no prominent effect on the *Muribaculaceae* or *Clostridia UCG-014*.

Spearman’s rank correlation was additionally applied to explore the associations between the differential bacterial taxa and host biochemical parameters, encompassing plasma lipid levels and hepatic oxidative stress indicators across all study cohorts (Figures 7C–F). Correlation matching between key differential bacterial species and biochemical indicators varied distinctly under different black pepper extracts intervention treatments. Under black pepper DES extract intervention, the corrected abundance of *Muribaculaceae*, *Clostridia* UCG-014, and other key differential species presented stable and regular correlation patterns with plasma lipid parameters (HDL-C, TG, LDL-C, TC) (Figure 7C) and hepatic oxidative stress indicators (SOD, MDA, GSH-Px) (Figure 7E), which was consistent with the improved metabolic and hepatic antioxidant status of acute ethanol-induced liver injury mice. In contrast, under black pepper ethanol extract intervention, the enriched *A. muciniphila* showed atypical correlation with oxidative stress indices (Figures 7D,F). Such distinct microbiota-biochemistry correlation profiles demonstrated that black pepper DES extract was associated with improvements in the perturbed gut microecology and microbiota-host metabolic interactions. Collectively, these results suggest that black pepper DES extract was associated with alterations in the composition of key gut bacterial species and may contribute to maintaining the homeostasis of the gut microbial community in mice with acute ethanol-induced liver injury.

## 4 Discussion

Medicinal and food plants represent a valuable reservoir for safe and effective hepatoprotective agents, and the gut microbiota is increasingly recognized as a key interface between dietary ingredients and host health (Samykutty et al., 2013; Derosa et al., 2016; Rathee et al., 2018). This study systematically optimized a choline chloride-1,4-butanediol DES ultrasonic-assisted extraction process for *P. nigrum* L. bioactives, and demonstrated that DES extract achieves comparable piperine yield to traditional ethanol extraction. The DES extract exhibits favorable *in vitro* antioxidant activity and *in vivo* hepatoprotective efficacy against acute ALD, suggesting a potential association with its gut microbiota-modulating capacity (Tian et al., 2025). These findings provide a sustainable green strategy for high-value utilization of plant-based spice resources, and scientific support for developing microbiota-targeted functional foods.

The optimized DES process achieved a piperine yield comparable to 60% ethanol extraction, consistent with previous reports showing that DESs exhibit excellent solubilization capacity for plant alkaloids due to their unique hydrogen bond network structure (Prabhune and Dey, 2023). Piperine was set as the extraction target, and HPLC was employed for qualitative analysis of the DES extract. The chromatogram confirmed the characteristic peak of piperine, indicating that the DES system is suitable for the extraction and detection of piperine from black pepper. In addition, several chromatographic peaks corresponding to unidentified components were observed alongside piperine. It is speculated that DES may exhibit broad-spectrum solubilization toward multiple phytochemical constituents in black pepper, which is consistent with the tunable solubility of DESs achieved by adjusting the types and ratios of hydrogen bond donors and acceptors, thereby altering their affinity for compounds of different polarities (Choi et al., 2011; Ma et al., 2025). However, the specific identities of these unidentified components require further investigation. Consistent with our prior work on *Lophatherum* gracile *Brongn.* flavonoid DES extraction (Luo et al., 2026), DES systems retain a broader spectrum of co-existing phytochemicals relative to ethanol extraction, yet the optimal DES matrix and dominant bioactive metabolites differ greatly between alkaloid and flavonoid plant sources.

Differential *in vitro* antioxidant performance of DES extract was further verified *in vivo* using an acute ethanol-induced liver injury mouse model. Excessive ethanol metabolism induces massive reactive oxygen species production in the liver, leading to lipid peroxidation, antioxidant enzyme depletion, and eventually hepatocellular damage and steatosis (Wang et al., 2023; Lee et al., 2025). In this study, both extracts alleviated ethanol-induced liver injury, as evidenced by reduced plasma transaminase levels, decreased hepatic lipid accumulation, and improved hepatic antioxidant enzyme activities. Of note, DES extract presented favorable hepatoprotective phenotypes in the ethanol-induced liver injury model. Such biological differences are correlated with its distinct *in vitro* antioxidant property and varied gut microbial composition, while definitive causal mechanisms require further verification.

Alcohol-associated liver disease progresses through distinct pathological stages, and multiple standardized mouse models have been established to recapitulate corresponding lesions. The well-documented chronic-plus-binge NIAAA model established by Bertola et al. (2013) exhibits high translational relevance for simulating clinical severe alcoholic hepatitis with prominent hepatic inflammatory infiltration. Nevertheless, we selected a 9-day repeated ethanol gavage model for the present work. Short-term ethanol exposure can reliably induce early gut-liver axis dysfunction and gut microbial dysbiosis, which matches our core research endpoints focused on preventive intervention against initial alcohol-related microecological disturbance. Besides, unified daily gavage ensures identical extract dosing across all groups, eliminating variable food intake bias inherent to liquid-diet protocols and enabling rigorous head-to-head comparison of two extraction products.

Accumulating evidence confirms that gut microbiota dysbiosis acts as a critical pathogenic driver in the development and progression of ALD (Sosnowski and Przybyłkowski, 2024). Ethanol exposure disrupts intestinal barrier integrity, induces overgrowth of pathobionts, and promotes translocation of bacterial endotoxins into the liver via the portal vein, further aggravating hepatic inflammation and injury (Chung et al., 2025). In this study, acute ethanol exposure induced significant gut microbiota dysbiosis in mice, characterized by marked enrichment of *Muribaculaceae* and *Clostridia* UCG-014 and decreased abundance of beneficial *L. murinus* and *A. muciniphila*. Consistent with our findings, Jin *et al.* also documented that *Muribaculaceae* was markedly enriched in the gut microbiota of mice following ethanol exposure (Jin et al., 2023). Notably, DES extract intervention effectively reversed ethanol-induced gut microbiota dysbiosis: it significantly inhibited overgrowth of *Muribaculaceae* and *Clostridia* UCG-014, restored *L. murinus* abundance, and reshaped the overall gut microbial community structure to near-normal statuses. *Lactobacillus murinus* is a well-documented beneficial gut microbiota that maintains intestinal barrier integrity by producing short-chain fatty acids and regulating tight junction proteins (Wu et al., 2026), and exerts anti-inflammatory and hepatoprotective effects via the gut-liver axis (Pan et al., 2024; Xie et al., 2024). Combined with Spearman correlation analysis, the key differential bacterial species modulated by black pepper DES extract exhibited stable and regular correlations with serum lipid profiles and hepatic oxidative stress indicators, forming a benign microbiota-host regulatory relationship. These distinct microbial regulatory profiles, coupled with coordinated shifts in lipid metabolism, suggest a potential association between DES-derived black pepper extract and microecological modulation. Such changes were accompanied by indicators consistent with intestinal barrier maintenance and hepatic metabolic adjustments. These observations warrant further investigation to determine whether these microbial and metabolic responses contribute to the attenuation of ethanol-induced liver injury (Yan et al., 2011; Mullish and Thursz, 2024; Zhang et al., 2025). Observed increase of *L. murinus* and *A. muciniphila* abundance may represent one contributing mechanism, although causality remains to be established.

This study has several limitations that warrant further investigation. First, given the complex multi-component nature of DES extracts, the specific active constituents responsible for enhanced hepatoprotective and microbiota-modulating effects remain to be isolated and identified (He et al., 2022). Further metabolomic analysis and component-activity validation are required to clarify the material basis (Lindell et al., 2022; Mohanty et al., 2024; Nie et al., 2024). Second, this study only verified phenotypic correlation between gut microbiota alteration and hepatic improvement. The causal relationship between specific bacterial taxa and protective effects, as well as downstream molecular pathways involved in gut-liver axis crosstalk, need to be further explored via fecal microbiota transplantation and germ-free mouse experiments. Third, the safety and stability of DES extracts in food processing and storage need systematic evaluation to promote industrial application.

## 5 Conclusion

In conclusion, this study successfully optimized a choline chloride-1,4-butanediol-based ultrasonic-assisted DES extraction process for *P. nigrum* L. bioactives. The optimized process achieves piperine yield comparable to traditional ethanol extraction and endows the extract with distinct *in vitro* antioxidant properties. In mice with acute ethanol-induced liver injury, DES extract intervention alleviated hepatic lipid accumulation, oxidative stress, and liver injury, and was associated with modulation of ethanol-induced gut microecological dysbiosis. Variations in gut microbiota composition were found to be associated with the hepatoprotective phenotypes of DES extract observed in this study. This work provides an efficient, sustainable green extraction strategy for high-value utilization of black pepper resources and offers preliminary insights for developing plant-derived hepatoprotective agents that correlate with gut-liver axis homeostasis.

## 6 Limitations

This study has several notable experimental limitations that should be acknowledged to contextualize the current findings and guide future research. First, liver index was not determined *in-vivo*, so we lack an auxiliary macroscopic marker for evaluating ethanol-triggered hepatomegaly and liver swelling injury. Second, although sequential macroporous-resin purification followed by dialysis was applied to remove DES components from DES extracts, we did not quantitatively measure residual choline chloride and 1,4-butanediol in final lyophilized samples. Moreover, a DES-only solvent control group (dialyzed DES without *P. nigrum* L. matrix) was absent. Thus, the hepatoprotective and gut-microbiota-reshaping phenotypes observed in the DES-extract group cannot be fully attributed to pepper-derived phytochemicals, and potential bioactive interference from residual DES cannot be completely excluded. Third, although piperine concentrations of the two lyophilized crude extracts were quantified (44.40 ± 0.84 mg/g for DES extract and 44.72 ± 0.54 mg/g for 60% ethanol extract), dry-extract yield per gram raw material and crude-drug equivalents were not documented. Animals received equal-mass doses (50 mg/kg) of whole crude extracts to compare the integrated bioactivity of products from two extraction processes. Despite the similar piperine content between preparations, subtle differences in actual piperine intake still existed. This dosing strategy enables comparison of overall extract performance but cannot fully disentangle the respective contributions of piperine versus other co-extracted phytochemical constituents. Fourth, the precision and recovery tests were not performed in the present study. Finally, only 16S rRNA gene sequencing t was performed to profile gut microbial composition, without measurement of key gut-liver-axis functional biomarkers. Therefore, we can only report statistical correlations between microbial taxa and hepatic biochemical indices, and direct functional evidence proving how DES extracts modulate the gut-liver axis is lacking. Collectively, these findings highlight multiple directions for future follow-up work, including supplementary quantitative pathological scoring, quantitative detection of residual DES components, complete HPLC method-validation (precision and recovery assays), and multi-omics analysis of gut microbial functional metabolites.

## Data Availability

The datasets presented in this study can be found in online repositories. The names of the repository/repositories and accession number(s) can be found below: https://www.ncbi.nlm.nih.gov/, PRJNA1504098.
